# Feasibility of Comparative Health Research Outcome of Novel Surgery in prostate cancer (IP4-CHRONOS): statistical analysis plan for the randomised feasibility phase of the CHRONOS study

**DOI:** 10.1186/s13063-021-05509-w

**Published:** 2021-08-18

**Authors:** Emily Day, A. Toby Prevost, Matthew R. Sydes, Deepika Reddy, Taimur T. Shah, Mathias Winkler, Tim Dudderidge, John Staffurth, Stuart McCracken, Vincent Khoo, Puja Jadav, Natalia Klimowska-Nassar, Thiagarajah Sasikaran, Hashim U. Ahmed, Francesca Fiorentino

**Affiliations:** 1 Imperial Clinical Trials Unit, School of Public Health, Imperial College London, Stadium House, 68 Wood Lane, London, W12 7RH UK; 2 Nightingale-Saunders Unit, King’s Clinical Trials Unit, King’s College London, London, UK; 3 MRC Clinical Trials Unit at UCL, University College London, London, UK; 4 Imperial Prostate, Division of Surgery, Department of Surgery and Cancer, Imperial College London, London, UK; 5 Imperial Urology, Charing Cross Hospital, Imperial College Healthcare NHS Trust, London, UK; 6 Department of Urology, University Hospital Southampton NHS Trust, Southampton, UK; 7 School of Medicine, Cardiff University, Cardiff, UK; 8 Department of Urology, Sunderland Royal Hospital, Sunderland, UK; 9 Faculty of Medical Sciences, Newcastle University, Newcastle upon Tyne, UK; 10 Department of Oncology, The Royal Marsden NHS Foundation and Institute of Cancer Research, London, UK; 11 Department of Surgery and Cancer, St Mary’s Hospital, Imperial College London, Queen Elisabeth the Queen Mother Building (10th Floor/1091), Praed Street, London, W2 1NY UK

**Keywords:** Focal therapy, Multi-centre multi-arm, Randomised controlled trial, Feasibility, Compliance, Recruitment

## Abstract

**Background:**

Randomised controlled trials (RCTs) for surgical interventions have often proven difficult with calls for innovative approaches. The Imperial Prostate (IP4) Comparative Health Research Outcomes of Novel Surgery in prostate cancer (IP4-CHRONOS) study aims to deliver level 1 evidence on outcomes following focal therapy which involves treating just the tumour rather than whole-gland surgery or radiotherapy. Our aim is to test the feasibility of two parallel RCTs within an overarching strategy that fits with existing patient and physician equipoise and maximises the chances of success and potential benefit to patients and healthcare services.

**Methods and design:**

IP4-CHRONOS is a randomised, unblinded multi-centre study, including two parallel randomised controlled trials targeting the same patient population: IP4-CHRONOS-A and IP4-CHRONOS-B.

IP4-CHRONOS-A is a 1:1 RCT and the other is a multi-arm, multi-stage (MAMS) RCT starting with three arms and a 1:1:1 randomisation. The two linked RCTs are discussed with patients at the time of consent and the choice of A or B is dependent on physician and patient equipoise. The primary outcome is the feasibility of recruitment, acceptance of randomisation and compliance to allocated arm.

**Results:**

This paper describes the statistical analysis plan (SAP) for the feasibility study within IP4-CHRONOS given its innovative approach. Version 1.0 of the SAP has been reviewed by the Trial Steering Committee (TSC), Chief Investigator (CI), Senior Statistician and Trial Statistician and signed off. The study is ongoing and recruiting. Recruitment is scheduled to finish later in 2021. The SAP documents approved methods and analyses that will be conducted. Since this is written in advance of the analysis, we avoid bias arising from prior knowledge of the study data and findings.

**Discussion:**

Our feasibility analysis will demonstrate if IP4-CHRONOS is feasible in terms of recruitment, randomisation and compliance, and whether to continue both A and B or just one to the main stage.

**Trial registration:**

ISRCTN ISRCTN17796995. Registered on 08 October 2019

## Background

Randomised controlled trials (RCTs) for surgical interventions have often proven difficult with calls for innovative approaches. The Imperial Prostate (IP4) Comparative Health Research Outcomes of Novel Surgery in prostate cancer (IP4-CHRONOS) study aims to deliver level 1 evidence on outcomes following focal therapy which involves treating just the tumour rather than whole-gland surgery or radiotherapy. Our aim was to test the feasibility of two parallel RCTs within an overarching strategy that fits with existing patient and physician equipoise. The study protocol has been published [1] [International Standard Randomised Controlled Trial Number: ISRCTN17796995].

Focal therapy targets individual areas of cancer within the prostate, to confer oncological control with minimal side-effects. It is an alternative approach to whole-prostate radical approaches such as radiotherapy or prostatectomy surgery. Evidence demonstrates encouraging short- and medium-term outcomes. However, there are currently no RCTs comparing focal therapy to radical therapies although a handful of National Health Service (NHS) UK centres offer it following National Institute of Health and Care Excellence (NICE) approval with special arrangements [2]. There has been concern that using a traditional framework for RCTs may be challenging to deliver randomised comparative data for focal therapy in localised prostate cancer given the numerous failures of RCTs in this disease space which look to compare different interventions [3]. One recent attempt was only partially successful with a requirement to decrease the target accrual and lengthen the time of the study [4], leading the investigators to use an investigational drug laser combination to deliver vascular targeted photodynamic (VTP) therapy which cannot be used outside of a trial framework in their follow-up main study to effectively ensure that patients wishing to have VTP focal therapy cannot access it in routine clinical care [5]. A general concern over surgical trials has been raised over the last decade with calls for innovative trial designs [6].

In this clinical practice context, to gain evidence of the effectiveness of focal therapy to treat patients with clinically significant cancer, two separate parallel RCTs, IP4-CHRONOS-A and IP4-CHRONOS-B, are being conducted. IP4-CHRONOS-A is a head-to-head RCT comparing focal therapy to radical radiotherapy/prostatectomy, and in parallel, in those who express a strong preference for focal therapy, we are conducting the first surgical multi-arm, multi-stage (MAMS) RCT (IP4-CHRONOS-B). A MAMS trial aims to answer multiple questions simultaneously under the same regulatory framework. In this type of design, multiple different treatment options can be compared simultaneously, often against a control arm [7]. One of the best-known examples of a MAMS trial is the STAMPEDE (Systemic Therapy in Advancing or Metastatic Prostate Cancer: Evaluation of Drug Efficacy) trial which looked at the treatment of men with advance or metastatic prostate cancer. For IP4-CHRONOS-B, this is a comparison of focal therapy alone to focal therapy combined with different neoadjuvant agents to determine whether failure can be improved with these additional treatments, starting by testing two commonly used hormonal agents, finasteride (5-alpha reductase inhibitor) or bicalutamide (anti-androgen) for 12 weeks in the lead up to the focal therapy using high-intensity focused ultrasound (HIFU) or cryotherapy. These ablative therapies are delivered under general anaesthetic in one session although can be repeated if there is evidence of residual or recurrent cancer.

Participation into IP4-CHRONOS-A or IP4-CHRONOS-B is determined by participant and physician preference and discussion. Because of the separate two parallel RCT design, the MAMS design and the patient and physician preference, it is essential to establish the feasibility of such a study within an overarching strategy that fits with existing patient and physician equipoise and maximises the chances of success and potential benefit to patients and healthcare services. The two linked RCTs will be discussed with patients and the choice of A or B will be dependent on physician and patient equipoise.

Not all centres across the UK offer focal therapy and therefore an important aspect of ascertaining feasibility is an estimate of the levels of equipoise between focal and radical therapy existing in those UK centres that do or do not offer focal therapy. Furthermore, as focal therapy is already offered in several centres in the UK under NICE Interventional Procedure guidance, some men and their physicians might have a strong preference for focal therapy.

We describe the statistical analysis plan (SAP) for the feasibility study. This ensures that the feasibility analysis is not data driven or selectively reported. This SAP was written following the guidelines for the statistical analysis plans by Gamble et al. [8]. Presentation of primary analyses is expected in late 2021, after all participants have been followed up for 3 months to measure compliance to the allocated arm. Results of the feasibility study will determine the deliverability and conduct of the main phase, pending further funding application.

## Methods and design

**Fig. 1:**
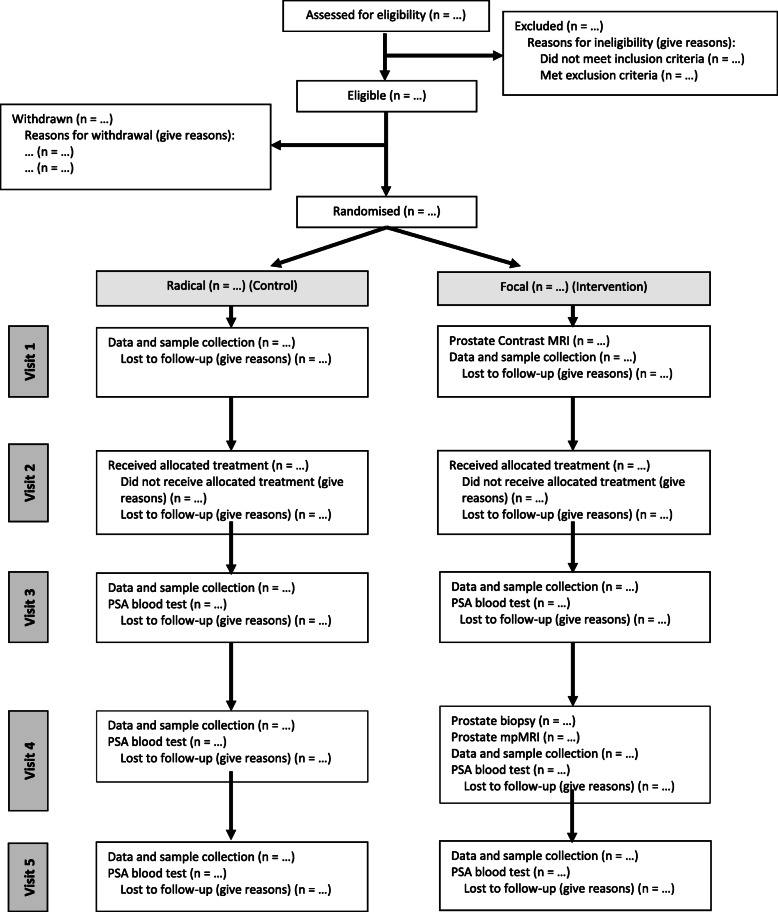
CONSORT diagram for IP4-CHRONOS-A

**Fig. 2:**
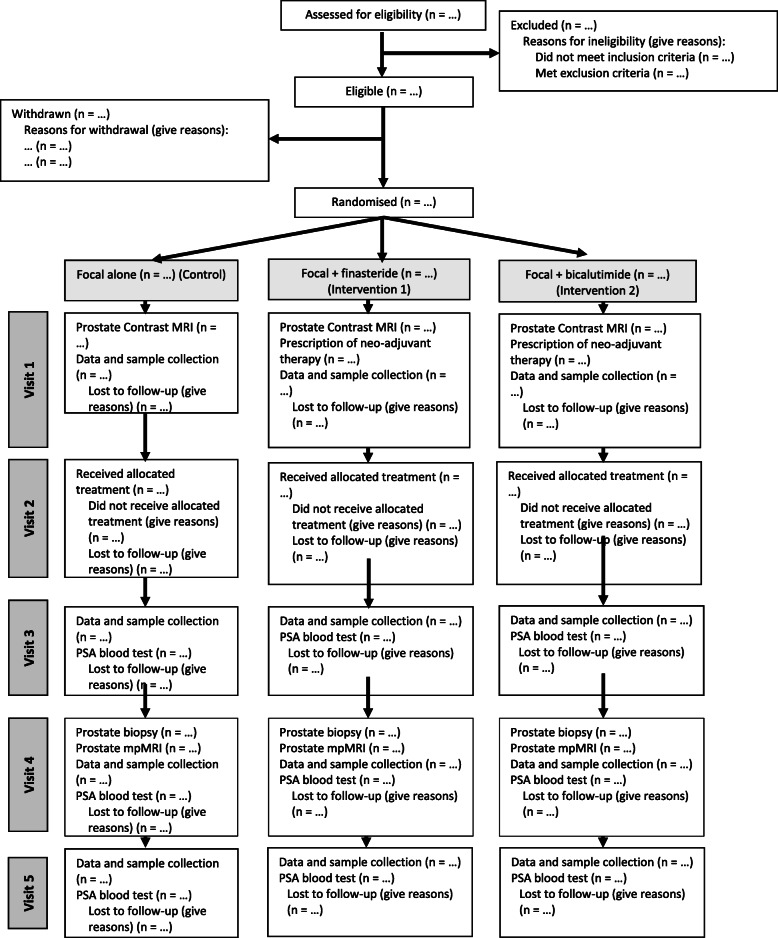
CONSORT diagram for IP4-CHRONOS-B

IP4-CHRONOS is a randomised, open-label multi-centre study, including two parallel RCTs targeting the same patient population. The comparator in both trials is standard of care. Consolidated Standards of Reporting Trials (CONSORT) diagrams are outlined for each study in Fig. 1 (IP4-CHRONOS-A) and Fig. 2 (IP4-CHRONOS-B).

**Fig. 3:**
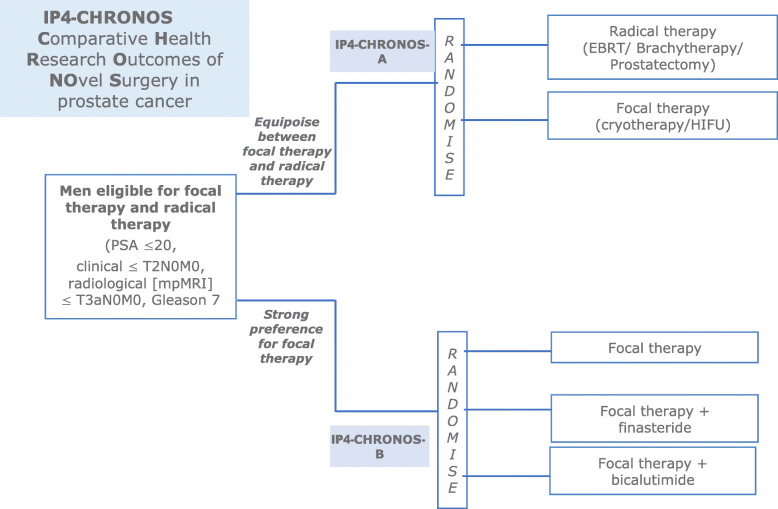
Study flowchart

The main phase of IP4-CHRONOS-A will be an open-label, two-arm, phase II/III non-inferiority RCT comparing standard of care radical therapy with focal therapy alone. To further facilitate accrual, the radical arm will align with patient and physician preferences and eligibility so that either radiotherapy or prostatectomy surgery can be chosen if patients are randomly allocated to the radical arm. The main phase of IP4-CHRONOS-B will be an open-label, three-arm, phase II/III MAMS RCT design comparing focal therapy with focal therapy plus neoadjuvant treatments: in the feasibility study, finasteride and bicalutamide. A summary of the treatment groups, for both IP4-CHRONOS-A and B, is presented in Table 1. Figure 3 and Table 2 present the study flowchart and visit schedule for both studies.

**Table 1:** Summary of treatment groups for the feasibility of IP4-CHRONOS-A and IP4-CHRONOS-B

Treatment sequence	Number of subjects	Details
**IP4-CHRONOS-A**		
Control arm	Feasibility, N=30	Radical radiotherapy or radical prostatectomy (as per physician and patient decision/preference)
Intervention arm	Feasibility, N=30	Focal therapy using HIFU or cryotherapy (as per physician and patient decision/preference)
***Total number***	Feasibility, N=60	
**IP4-CHRONOS-B**		
Control arm	Feasibility, N=20	Focal therapy using HIFU or cryotherapy (as per physician and patient decision/preference)
Intervention arm 1	Feasibility, N=20	Neoadjuvant finasteride 5mg once daily for a minimum of 12 weeks followed by focal therapy (as per standard care control arm for IP4-CHRONOS-B).
Intervention arm 2	Feasibility, N=20	Bicalutamide 50mg once daily for 12 weeks followed by focal therapy (as per standard care control arm for IP4-CHRONOS-B)
***Total number***	Feasibility, N=60

**Table 2:** Visit Schedule

	Screening & Consent	Visit*						
Visit	1	2	3	4	5	6	7	6 monthly visits until last visit 60 months after visit 2
Months			These below specify the months ***after*** the completion of each treatment in each arm					
		0	3	12	18	24	30	31-60. Visits 4 onwards can be telephone consultations in order to note clinical outcomes although MRI scans and biopsies where done will require physical visits to the hospital
Informed Consent and enrolment into either IP4-CHRONOS A or IP4-CHRONOS B	X							
Inclusion & exclusion criteria checked, including concomitant medication review	X							
Randomisation	X							
Prescription of neo-adjuvant therapy	X (if randomised to such arm)							Within 24hrs of randomisation
PSA blood test			X	X	X	X	X	X (6 monthly)
Prostate Contrast MRI	X (if randomised to focal therapy and no contrast given during diagnostic scan – to have prior to visit 2)							
Prostate mpMRI				X (focal therapy arms)				
Biopsy				X (focal therapy arms)				
Treatment		X (these vary in length)						X (focal therapy arms – a second treatment will be permitted for a histologically confirmed recurrent, residual or new out-of-field disease)
Clinical assessment (optional, only if required)			X	X	X	X	X	
PROMS questionnaires	X		X	X		X		X (every 12 months, at 24, 36, 48 and 60 months visits)
Review/ reporting of patient AEs/SAEs		X	X	X	X	X	X	X
Blood and urine tests including those for biobanking (optional)	X		X	X				X

*Time window for each visit will be +/- 4 weeks

Minimum length of follow up for the feasibility study will be 3 months from treatment for each patient. Then treatment will revert to standard of care

We will test what levels of equipoise exist in those UK centres that do or do not offer focal therapy, via a qualitative sub-study conducted by a team at Cardiff University. This analysis is not covered in the SAP.

### Study population

Patients with non-metastatic prostate cancer who are suitable for focal therapy and radical therapy will be approached for recruitment into IP4-CHRONOS.

Eligible patients will satisfy the following eligibility criteria.

#### Inclusion criteria

- Histologically confirmed prostate adenocarcinoma
- PSA ≤ 20 ng/ml
- Patients must have undergone a diagnostic pre-biopsy Magnetic Resonance Imaging (MRI) compliant with national uro-radiology consensus guidelines. Dynamic contrast enhancement using gadolinium is not required at the diagnostic stage. However, contrast enhancement MRI will be required in those men who undergo focal therapy prior to focal therapy as a baseline for comparison during follow-up. In the absence of a compliant diagnostic magnetic resonance imaging (MRI) (for clinical or other reasons), a transperineal template mapping biopsy using a 5-10 mm sampling frame will be required
- Overall Gleason score of 7 (either 3 + 4 = 7 or 4 + 3 = 7) of any length or Gleason 3 + 3 = 6 provided ≥ 6mm cancer core length in any one core. Patients with Gleason 4 + 4 = 8 in some cores but where the overall Gleason score is 7 will be included
- Patients with bilateral histologically proven prostate cancer are permissible provided the following criteria are met:
  - ◦ The index lesion to be treated, if focal therapy is used, meets the above histological criteria
  - ◦ The patients may have a Prostate Imaging Reporting and Data Systems (PIRADS) or Likert score 3, 4 and 5 multi-parametric Magnetic Resonance Imaging (mpMRI) lesion in the same hemi-gland (either right/left or anterior/posterior) as the histological index lesion
  - ◦ Secondary areas of Gleason 3 + 3 = 6 of ≤ 5mm cancer outside of the treatment field can be monitored, if present, and the patient undergoes focal therapy
  - ◦ If a Likert or PIRADS score 3, 4 or 5 mpMRI lesion is present in an area outside of the treatment field, with a negative biopsy for cancer, then pathology must be reviewed with confirmation of the presence of inflammation or atrophy, if the patient is to undergo focal therapy*
- Radiological stage T2b/T3a will require central review regarding suitability for focal therapy
- Index tumour volume, as seen on multi-parametric MRI (mpMRI) if carried out, will be restricted to 50% of one lobe for with for either unilateral or bilateral ablation. Patients with tumour volume >/= 50% of one lobe will require central review prior to enrolment. Final decisions on the suitability of focal therapy will lie with the trial central review in these cases**
- Age at least 18 years of age
- Participants must be fit to undergo all procedures listed in the protocol as judged by the clinical team

*A biopsy of a suspicious mpMRI area may miss underlying cancer due to targeting error. However, if there is an alternative diagnosis for the changes on mpMRI such as inflammation or atrophy, then this risk is reduced.

**This is to ensure that inappropriately large tumours are not being treated with focal therapy.

#### Exclusion criteria

- Previous or current LHRH agonist or LHRH antagonist or anti-androgen use in IP4-CHRONOS-B
- Patients already established on a 5-alpha reductase inhibitor (finasteride or dutasteride) who wish to go into IP4-CHRONOS-B will need to discontinue this for at least 6 months prior to randomisation (NB: testosterone supplementation is permitted)
- Previous treatment for prostate cancer
- Life expectancy likely to be less than 10 years
- Unable to give informed consent

The patients are first identified at the multidisciplinary team (MDT) and then both A and B are discussed with the patient during their appointments. An MDT is a group of professionals from one or more clinical disciplines who together make decisions regarding recommended treatment of individual patients. All patients will be offered both studies with those in equipoise between focal and radical therapy potentially agreeing to participate in IP4-CHRONOS-A and those expressing a preference for focal treatment (not in equipoise between focal and radical therapy), potentially participating in IP4-CHRONOS-B. The patient will be called or emailed after a minimum of 24 h and asked if they would like to take part. If they agree, remote consent will be taken, and randomisation within the chosen study will be performed.

### Sample size

The aim of the feasibility study is to recruit 120 patients from at least 6 centres over 12 months for both A and B (60 patients in each). Sixty participants per trial will allow an estimate of recruitment rate of 33% with a 95% confidence interval of [0.211, 0.449].

The target sample size for IP4-CHRONOS-A was adjusted because of centre opening problems and allocated resources to the study due to the Coronavirus Disease 2019 (COVID-19) pandemic. The minimum number of participants required to assess the IP4-CHRONOS-A feasibility of recruitment is 36 in 8 months (recruitment rate of 33% ± 15%). The maximum number of participants remains at 60 as per the study sample size calculation.

### Randomisation

Randomisation is blocked and stratified by the following stratification factors:

- Tumour grade (Gleason 6 [grade group 1], Gleason 7 [grade group 2], Gleason 7 [grade group 3])
- Local stage (T2 versus radiological (MRI) T3)
- Previous or current 5-alpha reductase inhibitor use (for A only)

### Feasibility study objectives

The feasibility objective for IP4-CHRONOS-A is to determine if patients agree to participate in an RCT that randomly assigns them to focal therapy alone or radical therapy (radiotherapy or prostatectomy). The feasibility objective for IP4-CHRONOS-B is to determine if patients expressing a preference for focal therapy agree to participate in a multi-arm, multi-stage (MAMS) RCT that randomly assigns them to focal therapy alone or focal therapy in combination with neoadjuvant and/or adjuvant agents.

## Study outcomes

### Primary outcome measures

The primary outcome measures for both A and B are the feasibility of recruitment, acceptance of randomisation and compliance to allocated arm. The feasibility of recruitment will be determined by recruitment rates to each study. Recruitment rate is defined as the total number of patients recruited (consented) out of the total number of patients approached. Randomisation rate is defined as the total number of patients randomised out of the total number of patients recruited (consented). Recruitment and randomisation rates will be calculated for A and B separately. Recruitment to the study is defined as the patient giving informed consent, and so the date of recruitment is equal to the date of informed consent.

Compliance comprises treatment compliance and drug compliance. Treatment compliance is measured in both A and B. Treatment compliance is defined as the proportion of patients who underwent treatment as randomly allocated. Drug compliance is only measured in IP4-CHRONOS-B for those patients who are randomly allocated to receive focal therapy plus a neoadjuvant drug treatment. Drug compliance is defined using two definitions: the proportion of patients who return their empty blister packs and the proportion of patients who are given the neoadjuvant drug and who do not have a registered protocol deviation (stating that the drug was taken for less than 8 weeks). Both estimates for drug compliance will be presented, and the Trial Management Group, independent Trial Steering Committee (TSC) and future funders will determine what constitutes feasibility of compliance. Participants who drop out of the study before compliance data has been collected will be recorded as missing and will not be included in the compliance analysis.

### Secondary outcome measures

Patients’ experience of each treatment arm including systemic issues, erectile dysfunction, urinary symptoms and rectal symptoms will be summarised using their responses to Patient Reported Outcome Measures (PROMS): EQ-5D-5L, IIEF-15, EPIC-26 and EPIC-Urinary Domain. These will be investigated using frequency tables and graphical visualisations at the relevant time points.

## Statistical determination of feasibility

### Recruitment rate

For each of IP4-CHRONOS-A and IP4-CHRONOS-B, progression to the main stage will be deemed appropriate if the recruitment rate is above 21.1% (lower end of the confidence interval for recruitment rate of 33% for 60 patients).

If the overall recruitment rate, estimated at the end of the feasibility study, is below 21.1%, this will suggest that the trial would have low recruitment feasibility and, after completion of patient follow-up for a minimum of 3 months, should not proceed to the main stage. The results will be presented to the sponsor, Trial Management Group, the TSC and future funders. If the recruitment rate is between 21.1 and 33%, then identifiable remedial work to improve the recruitment rate will be implemented in the main phase of the trial. If the recruitment rate is greater than or equal to 33%, the trial will be deemed feasible.

If the trial does not proceed to the main stage following the analysis of the feasibility study, patients will still be followed up for a minimum of 3 months and will then revert to standard of care in which the clinical care provided to patients will not differ from the clinical follow-up stipulated in the protocol. At the end of the study, patients will continue to be followed up locally within their recruitment centres with the ICE (European Registry for Cryosurgical Ablation of the prostate, EuCAP) or the HEAT international HIFU registry as per NICE guidelines IPG432/IPG42.

### Treatment compliance

For each of IP4-CHRONOS-A and IP4-CHRONOS-B, if the lower end of the confidence interval for the proportion of patients who underwent treatment is ≥ 80%, then we have viable compliance to progress to the main phase. If the lower end of the confidence interval for the proportion of patients who underwent treatment is between 70 and 80%, then this indicates that identifiable remedial work might be needed to improve this for the main stage. If the lower end of the confidence interval for the proportion of patients who underwent treatment is < 70%, then this could affect the primary outcome analysis and threaten the validity of the study.

### Drug compliance

For IP4-CHRONOS-B only, if the lower end of the confidence interval for the proportions of patients who complied to taking the neoadjuvant drug is between 80 and 90%, then this indicates that identifiable remedial work may be needed to improve this for the main stage. If the lower end of the confidence interval for the proportions is < 80%, then this could affect the primary outcome analysis and threaten the validity of the study and may require sample size re-estimation.

### Embedded qualitative component

The integrated qualitative component is designed to inform the primary and secondary trial objectives in the trial recruitment and testing stages. Participant interview data highlighting trial processes in need of improvement may be used in real time to allow timely protocol amendments to improve recruitment and retention of participants. Healthcare professionals (physicians, nurses) responsible for recruiting patients will also be interviewed.

A data analysis plan of the qualitative component has been written, and the analysis will be conducted by the team at the School of Medicine at Cardiff University. Anonymised transcripts will be analysed using deductive thematic analysis techniques, with a coding framework developed to reflect the trial outcomes. Analysis will begin with two qualitative researchers individually coding the first three interview transcripts. The data sets will be coded in full and organised into themes and subthemes. This data will be presented in a narrative format and interpreted within the context of patient experience to inform outcomes and reflect the aims of the trial.

## Analysis principles

All feasibility outcomes will include all patients recruited (consented) to the trials (IP4-CHRONOS-A and B) during the feasibility phase, by arm.

Summaries of continuous variables will be presented as means and standard deviations if approximately normally distributed, and as medians and inter-quartile ranges for skewed data; categorical variables will be presented as frequencies and percentages. Normality of continuous variables will be checked visually by plotting the data and inspecting the distribution. Proportions calculated as part of the feasibility outcomes analyses will be presented along with corresponding 95% confidence intervals. A 5% significance level will be used.

Baseline characteristics will be summarised by trial and by arm. These include demographics (age, ethnicity, Index of Multiple Deprivation (IMD) decile), Digital Rectal Examination results, details of current medications, International Prostate Symptom Score (IPSS) score, and Maximum Cancer Core Length (MCCL) and Gleason grade at pre-enrolment biopsy. Baseline characteristics will also be summarised for those who withdrew and those who completed each trial.

### Primary outcome analysis summary

The total number of patients recruited to IP4-CHRONOS will be calculated and reported. Also, the number of patients recruited to A and B will be reported to estimate the feasibility for each RCT.

The primary analysis of the feasibility study will calculate the mean number of patients recruited and randomised per month, per centre (including only the months in which the centre is open and recruiting). Overall recruitment and randomisation rates will also be calculated, along with their corresponding 95% confidence intervals. Graphs displaying the recruitment and randomisation rates over time will be presented. A graph displaying the actual versus target recruitment rates will also be presented.

The proportions of patients in each treatment arm who underwent treatment will be presented, along with their corresponding 95% confidence intervals. Treatment compliance will be evaluated by arm and by each RCT (A or B). Viable treatment compliance is defined as the lower end of the confidence interval for the proportion of patients who underwent treatment being ≥ 80%.

Neoadjuvant drug treatment in IP4-CHRONOS-B is prescribed at randomisation. The proportions who returned their empty blister packs, and the proportions who took the drug and did not have a registered protocol deviation, stating that they took the drug for less than 8 weeks, in each treatment arm will be reported, along with their corresponding 95% confidence intervals. Drug compliance will be evaluated by arm for each of the drug compliance definitions. Viable drug compliance is defined as the lower end of the confidence interval for the proportions being ≥ 90%.

Other analyses for the primary outcomes include Kaplan-Meier curves presenting the time from randomisation to withdrawal, summary statistics of time between key events throughout the trial (consent, randomisation, treatment, neoadjuvant drug prescription, visits) and safety (adverse and serious adverse events). Kaplan-Meier analysis will be reported using the KMunicate format (https://www.ctu.mrc.ac.uk/our-research/methodology/conduct/kmunicate/) which includes displaying the uncertainty around the survival curves and risk tables for the number of patients at risk at each time point. Safety data will be reported in line with the Imperial Clinical Trials Unit (ICTU) standard operating procedures.

Reported adverse events (AEs) and serious adverse events (SAEs) will be listed and then summarised, by treatment arm, in terms of severity grade (using v4.0 Common Terminology Criteria for Adverse Events (CTCAE) grading) and causal relationship to treatment, for IP4-CHRONOS-A and B separately.

### Secondary outcome analysis summary

Patient-reported outcome measures (PROMs) are collected at baseline and 3 months. These include EQ-5D-5L, IIEF-15, EPIC-26 and EPIC-Urinary Domain. These analyses will be conducted for IP4-CHRONOS-A and IP4-CHRONOS-B.

EQ-5D-5L has five dimensions: mobility, self-care, usual activities, pain/discomfort and anxiety/depression, each measured using five levels of response [9]. Summary statistics, by treatment arm, will be presented for each of the dimensions and levels, at each time point. Histograms will also be produced to display the proportions in each level for each dimension.

EQ visual analogue scale (VAS) is an additional question on the EQ-5D-5L [9]. This will be summarised, by treatment arm, at each time point.

IIEF-15 consists of five domains: erectile function, orgasmic function, sexual desire, intercourse satisfaction and overall satisfaction [10]. EPIC-26 consists of five domains: urinary incontinence, urinary irritative/obstructive, bowel, sexual and hormonal [11]. EPIC-Urinary Domain consists of four subscales: function, bother, incontinence and irritative/obstructive [11]. Response score will be standardised and summary statistics, by treatment arm, will be presented for each domain, at each time point.

## COVID-19 adjustment

On 05/03/2020, COVID-19 was added to Public Health England’s list of notifiable diseases in England and Wales [12]. This date will be used as a cut-off to define before and during/after the COVID-19 pandemic analysis populations. Primary outcome analysis will be adjusted for the COVID-19 pandemic.

Primary outcome analysis concerning recruitment and randomisation will be calculated, separately, for those patients who were randomised before and during/after the COVID-19 pandemic. Primary outcome analysis concerning treatment compliance will be calculated, separately, for those patients who started treatment before and during/after the COVID-19 pandemic. Drug compliance analysis will be calculated, separately, for those patients who were randomly allocated to focal plus neoadjuvant drug treatment (finasteride or bicalutamide) in IP4-CHRONOS-B and started their neoadjuvant drug treatment before and during/after the COVID-19 pandemic.

Summary statistics of time between key events throughout the trial (consent, randomisation, treatment, neoadjuvant drug prescription, visits) will be calculated for patients whose most recent event is before and during/after the COVID-19 pandemic. Reasons for withdrawal will be reported for patients who withdrew from the study before and during/after the COVID-19 pandemic.

These analyses will be presented if there exist patients in both the before and during/after COVID-19 analysis populations. Analyses for populations which do not contain any patients will be omitted as they are already included in the main study analysis.

COVID-19-related protocol deviations will be summarised alongside other protocol deviations for the study.

## Missing data and outliers

A specific missing data mechanism is not required for the feasibility study. No formal method will be used for handling outliers.

## Software details

STATA v17 (or above) will be used for all analyses.

## Conclusion

IP4-CHRONOS provides an innovative trial design in what is recognised as a difficult-to-recruit disease space, localised prostate cancer, especially given the surgical interventions involved. The study attempts to match patient and physician equipoise and might provide valuable insights into whether such an overarching strategy might provide some solutions to the ongoing problems we have had in delivering randomised comparative trials in these areas. Our planned analysis strategy for the feasibility phase has been set out here to reduce the risk of reporting bias and data-driven analysis. Any deviations from the methods described in this paper will be detailed and justified fully in the final statistical report.

## Data Availability

Not applicable.

## Abbreviations

AE: Adverse event
CI: Chief Investigator
CONSORT: Consolidated Standards of Reporting Trials
COVID-19: Coronavirus disease 2019
CTCAE: Common Terminology Criteria for Adverse Events
HIFU: High-intensity focused ultrasound
IMD: Index of Multiple Deprivation
IP4-CHRONOS: Imperial Prostate (4) Comparative Health Research Outcome of Novel Surgery in prostate cancer
IPSS: International Prostate Symptom Score
ISRCTN: International Standard Randomised Controlled Trial Number
MAMS: Multi-arm, multi-stage
MCCL: Maximum Cancer Core Length
MDT: Multidisciplinary team
mpMRI: Multi-parametric magnetic resonance imaging
MRI: Magnetic resonance imaging
NICE: National Institute of Health and Care Excellence
NHS: National Health Service
PIRADS: Prostate Imaging and Reporting Data System
PROMs: Patient-reported outcome measures
RCT: Randomised controlled trial
REC: Research Ethics Committee
SAE: Serious adverse event
SAP: Statistical analysis plan
STAMPEDE: Systematic Therapy in Advancing or Metastatic Prostate cancer: Evaluation of Drug Efficacy
TSC: Trial Steering Committee
UK: United Kingdom
VAS: Visual analogue scale
VTP: Vascular targeted photodynamic
